# Supplementary material for: Identification of Novel Biomarker for Early Detection of Diabetic Nephropathy
Source: Biomedicines. 2021 Apr 22;9(5):457. doi: 10.3390/biomedicines9050457 (PMC8146473; doi:10.3390/biomedicines9050457)
Supplement: Supplementary file 1 [file biomedicines-09-00457-s001.zip › biomedicines-1183389-supplementary (1).pdf]

biomedicines

Identification of Novel Biomarker for Early Detection of Diabetic Nephropathy

Supplementary Materials

One supplementary figure and four supplementary tables.

Table S1. Sequences of primers used in PCR analysis.

Gene	5' to 3'	3' to 5'
Cxcr6	AGGCACCTATGAGTGGGTCT	TTGAAGGCCTTGG-TAGCCTG
Cfd	CTGCATGGATGGAGTGACCA	CCGGGTGAAGCAC-TACACTT
C4b	CTTTCTGGTCCGGGCTTCTT	TACTGGGGGTCTCCCTT-GAG
Lif	GTGTCCCCGACAACTCTAGC	GGACCACCGCAC-TAATGACT
Gapdh	ATCGACCACTACCTGGGCAA	AGGATAACGCAGGCGATGT

Table S2. List of secreted protein associated with kidney injury (5 W only up-regulated). (FDR < 0.05, Log fc >1.5).

Gene Symbol	Gene Name	Fold-Change
Kif18b	Kinesin family member 18B	2.29
Mmp9	Matrix metallopeptidase 9	1.80
Ccna1	Cyclin A1	1.56

Table S3. Common upregulation of secreted genes associated with kidney injury (FDR < 0.05, Log fc >1.5).

Gene Symbol	Gene Name	Fold-Change	
		5 Week	12 Week
Havcr1	hepatitis A virus cellular receptor 1	6.02	6.81
Fgg	fibrinogen gamma chain	3.96	3.83
Fgb	fibrinogen beta chain	3.02	2.80
Birc5	baculoviral IAP repeat-containing 5	2.80	1.96
Cdk1	cyclin-dependent kinase 1	2.79	1.96
Lcn2	lipocalin 2	2.59	4.98
Lif	leukemia inhibitory factor	2.59	3.93
Spp1	secreted phosphoprotein 1	2.59	3.85
Ube2c	ubiquitin-conjugating enzyme E2C	2.50	1.85
Atf3	activating transcription factor 3	2.36	4.11
Mcm5	minichromosome maintenance complex component 5	2.14	1.64
Serpine1	serpin family E member 1	1.88	3.66
Sox11	SRY box 11	1.86	1.55

Table S4. List of secreted protein associated with kidney injury (12 W only up-regulated). (FDR < 0.05, Log fc >1.5).

Gene Symbol	Gene Name	Fold-Change
Postn	Periostin	4.20
C4b	complement component 4B	3.88
Ccl2	C-C motif chemokine ligand 2	3.62
Clu	clusterin	3.58
Cxcl1	chemokine (C-X-C motif) ligand 1	3.36
Foxp3	forkhead box P3	3.22
Alox15b	arachidonate 15-lipoxygenase, type B	3.18
C4a	complement component 4A	3.15
C3	complement component 3	2.79
Adamts1	ADAM metallopeptidase with thrombospondin type 1 motif, 1	2.78
Cxcr6	chemokine (C-X-C motif) receptor 6	2.68
CFD	complement factor D	2.66
Tnfrsf12a	tumor necrosis factor receptor superfamily, member 12a	2.58
Mrc1	mannose receptor, C type 1	2.51
Cd3g	CD3g molecule	2.50
Bcl3	B-cell CLL/lymphoma 3	2.47
Epdr1	ependymin related 1	2.33
Lgals3	lectin, galactoside-binding, soluble, 3	2.28
Hspa1a	heat shock 70kD protein 1A	2.24
Timp1	TIMP metallopeptidase inhibitor 1	2.23
Sdc1	syndecan 1	2.22
C6	complement component 6	2.21
Tf	transferrin	2.15
Faslg	Fas ligand	2.14
Fn1	fibronectin 1	2.13
Fstl3	follistatin like 3	2.12
Cp	ceruloplasmin (ferroxidase)	2.11
Galnt6	polypeptide N-acetylgalactosaminyltransferase 6	2.11
Cd68	Cd68 molecule	2.07
Crispld1	cysteine-rich secretory protein LCCL domain containing 1	2.06
Cd14	CD14 molecule	2.05
S100a4	S100 calcium-binding protein A4	2.04
Il7	interleukin 7	2.03
Igfbp1	insulin-like growth factor binding protein 1	2.02
C1qb	complement component 1, q subcomponent, B chain	2.02
Cd27	CD27 molecule	2.02
Capg	capping actin protein, gelsolin like	2.00

Table S4. (continued).

Gene Symbol	Gene Name	Fold-Change
Cd276	CD276 molecule	1.99
Rprm	Reprimo, TP53 dependent G2 arrest mediator homolog	1.98
Elf3	E74 like ETS transcription factor 3	1.98
Chi3l1	Chitinase 3 like 1	1.95
Siglec5	Sialic acid binding Ig like lectin 5	1.92
Il1b	Interleukin 1 beta	1.91
Cntn1	Contactin 1	1.91
Nefh	Neurofilament heavy	1.90
Eps8l3	EPS8 like 3	1.86
Lyz2	lysozyme 2	1.83
C2	Complement C2	1.81
Cd84	CD84 molecule	1.81
Clcf1	Cardiotrophin like cytokine factor 1	1.79
Plekhs1	Pleckstrin homology domain containing S1	1.79
Anxa2	Annexin A2	1.77
Rab27b	RAB27B, member RAS oncogene family	1.77
Axl	AXL receptor tyrosine kinase	1.75
Mmp14	Matrix metallopeptidase 14	1.75
Anxa1	Annexin A1	1.73
Chac1	ChaC glutathione specific gamma-glutamylcyclotransferase 1	1.70
Cldn3	Claudin 3	1.69
Creb5	CAMP responsive element binding protein 5	1.69
Icam1	Intercellular adhesion molecule 1	1.68
Cd24	CD24 molecule	1.64
Hbegf	Heparin binding EGF like growth factor	1.63
Pdlim7	PDZ and LIM domain 7	1.63
Penk	Proenkephalin	1.62
S100b	S100 calcium binding protein B	1.61
Tnf	Tumor necrosis factor	1.60
Spi1	Spi-1 proto-oncogene	1.59
S100a9	S100 calcium binding protein A9	1.59
Samsn1	SAM domain, SH3 domain and nuclear localization signals 1	1.58
Dkk2	Dickkopf WNT signaling pathway inhibitor 2	1.57
Ctgf	Connective tissue growth factor	1.56
Cxcl10	C-X-C motif chemokine ligand 10	1.56
Dkk3	Dickkopf WNT signaling pathway inhibitor 3	1.53
Prcp	Prolylcarboxypeptidase	1.50
Vim	Vimentin	1.50

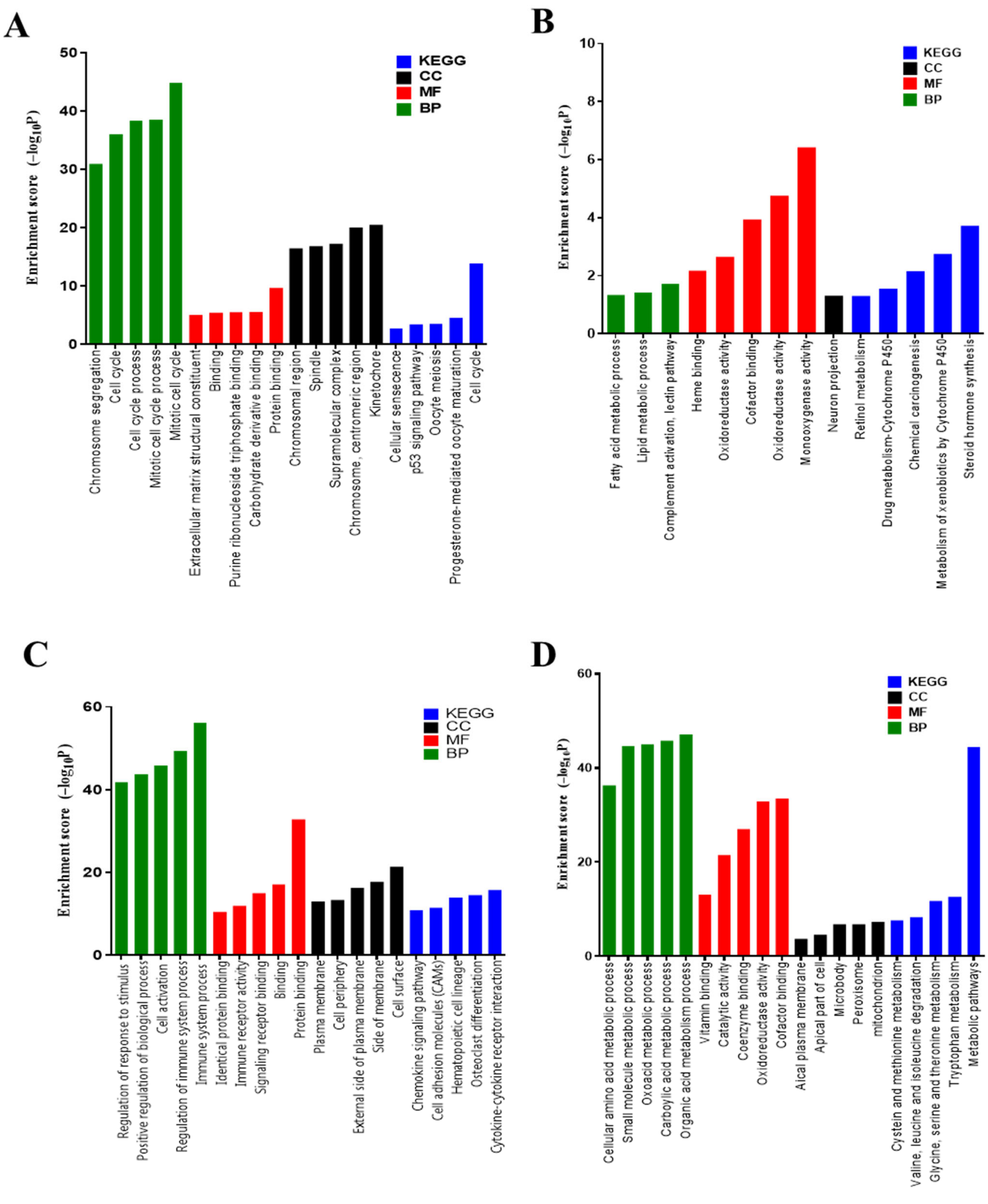

Figure S1. Functional and pathway enrichment analysis of DEGs among the different groups of rats. GO and KEGG enrichment analysis of up-regulated DEGs in 5 weeks (**A**) and 12 weeks (**C**) and down-regulated DEGS in 5 weeks (**B**) and 12 weeks (**D**) high-fat diet rats compared with non-diabetic rats. GO results were categorized into three parts: biological process (green), cellular component (black), and molecular function (red). KEGG results were categorized into 5 main categories.
